# Preparation and Characterization of a Novel Natural Quercetin Self-Stabilizing Pickering Emulsion

**DOI:** 10.3390/foods12071415

**Published:** 2023-03-27

**Authors:** Shenglan Lu, Xueying Li, Xunran Wei, Caihuan Huang, Jie Zheng, Shiyi Ou, Tao Yang, Fu Liu

**Affiliations:** 1 Department of Food Science and Engineering, Jinan University, Guangzhou 510632, China; 2 School of Pharmacy, Hainan Medical University, Haikou 571199, China

**Keywords:** quercetin, Pickering, antisolvent precipitation, crystalline

## Abstract

In contrast to their well-known physiological properties, phytochemicals, such as flavonoids, have been less frequently examined for their physiochemical properties (e.g., surface activity). A natural quercetin self-stabilizing Pickering emulsion was fabricated and characterized in the present study. The antisolvent precipitation method was used to modify quercetin (in dihydrate form), and the obtained particles were characterized by light microscope, atom force microscope, XRD, and contact angle. The antisolvent treatment was found to reduce the particle size, crystallinity, and surface hydrophobicity of quercetin. We then examined the effects of the antisolvent ratio, particle concentration, and oil fraction on the properties of the quercetin particle-stabilized emulsions. In addition, increasing the antisolvent ratio (1:1~1:10) effectively improved the emulsification performance of the quercetin particles. The emulsion showed good storage stability, and the particle size of the emulsion decreased with the rising particle concentration and increased with the rising oil phase ratio. The findings indicate that natural quercetin treated with antisolvent method has a good ability to stabilize Pickering emulsion, and this emulsion may have good prospective application potential for the development of novel and functional emulsion foods.

## 1. Introduction

Due to their surfactant-free nature, high stability, and good delivery capacity, Pickering emulsions, or particle-stabilized emulsions, have good prospects for application in food and pharmaceutical fields [1,2,3,4,5,6,7,8,9,10,11,12]. Traditional Pickering emulsions are mostly stabilized by inorganic particles, such as silica, which are limited in applications in food and other industries. Therefore, the search for more natural and safe particle stabilizers with good compatibility is one of the hot research topics in recent years in the field of food colloids, etc. [3,6,8,13]. Through their material source, the particles that have been reported to stabilize food-grade Pickering emulsions are mainly protein-based particles, polysaccharide-based particles, and other particles. These particles, however, are highly variable in nature, usually need to be modified or compounded, and mainly serve a single purpose. As a result, the search for food grade particle stabilizers with more sustainability and functionality are still ongoing. 

In addition to their well-known physiological properties, natural phytochemicals, such as flavonoid, have also been found to stabilize Pickering emulsions [14,15]. Murray et al. previously discovered that different types of natural flavonoids could stabilize oil-in-water Pickering emulsions and further investigated the effect of pH on emulsions properties [16,17]. They then found that water-insoluble natural polyphenols (quercetin and curcumin) dispersed in the oil phase could stabilize water-in-oil Pickering emulsions, and that the addition of protein emulsifiers in the aqueous phase could have a synergistic stabilizing effect [18,19]. Nanosized natural substances can also be used for Pickering emulsion stabilization. Typically, there are two kinds of methods used to reduce materials into nanosized particles: top-down and bottom-up [20]. The top-down method often involves a high energy input, such as high-pressure homogenization and wet milling technology [21,22,23]. Zhang et al. found that puerarin nanocrystals obtained using high pressure homogenization can stabilize oil-in-water Pickering emulsions [24]. Wang et al. reported that quercetin nanocrystals obtained by high pressure homogenization could be used for the stabilization of oil-in-water Pickering emulsions [25]. Silybin nanocrystals with 340 nm were also fabricated using high-pressure homogenization methods under >80 MPa [26]. This method often requires a high energy input and only reduce the particles size, without significantly changing the surface properties. In contrast to high-pressure homogenization, antisolvent precipitation or solvent volatilization is a low energy method (bottom-up) that can not only reduce the particle size, but also potentially change the surface properties of the particles [27,28,29]. Aditya et al. obtained amorphous curcumin nanoparticles using an antisolvent precipitation process and successfully used them for the stabilization of Pickering emulsion [30]. Liu et al. also used the antisolvent precipitation method to treat phytosterols, which was successfully used for the stabilization of water-in-oil Pickering emulsions [31,32]. Compared with the particles obtained by high-pressure homogenization, the antisolvent precipitation method has the advantages of being energy saving and easy to change the surface properties of the particles. At present, this technology is mainly used for the preparation of bioactive substances for nano-sizing and delivery. This technique has not been fully investigated for the preparation of food-grade nanoparticles (especially phytochemical-based) for the stabilization of Pickering emulsions.

Quercetin is a flavonoid compound widely found in vegetables and fruits, which has antioxidant, anti-inflammatory, antiviral, and cardiovascular protective effects [33,34,35]. However, the low solubility, chemical instability, and low bioavailability of quercetin in water limit their many applications [36,37,38,39,40,41]. Many scholars have designed delivery systems (e.g., emulsions, liposomes, nanoparticles, etc.) to solve this problem, but they often suffer from a low drug loading capacity and extensive use of surfactants [36,42,43,44,45,46,47,48,49,50]. In the studies of bioactives such as quercetin, the main focus has been on their physiological functions and how they potentiate, with less attention paid to their physicochemical properties (amphiphilicity). The inherent crystalline nature of flavonoids makes them excellent candidates for stabilizing Pickering emulsions (particle-stabilized emulsions) if they have proper surface wettability. Although some recent studies have successively found that flavonoids can stabilize Pickering emulsions, most of these flavonoids are used in their native state or are high-pressure processed [17,18,25]. The effect of antisolvent precipitation on the performance of flavonoids (e.g., quercetin) in Pickering emulsion stabilization have been less frequently studied.

In this paper, quercetin was treated by an antisolvent precipitation method with low energy consumption and the properties of its emulsion were investigated. Firstly, the quercetin particles obtained by antisolvent precipitation were characterized in terms of their morphology, size, crystallinity, and contact angle. Secondly, the basic properties of the above quercetin particle-stabilized emulsions, including the type of emulsion, particle size, and microstructure, were investigated.

## 2. Materials and Methods

### 2.1. Materials

The quercetin (dihydrate, >97%) was purchased from Shanghai Aladdin Reagent Co., Ltd. (Shanghai, China). The soybean oil was purchased from a local supermarket in Guangzhou (China). All of the other reagents were analytically pure.

### 2.2. Antisolvent Precipitation Treatment for Quercetin

Quercetin was dissolved in anhydrous ethanol (1%, *w*/*v*) and kept at 45 °C for 5 min. The organic phase containing quercetin was added to the aqueous phase (10 mM phosphate buffer, pH = 7.0) within 1 min using a high-speed homogenizer (IKA T25 digital ultraturrox, IKA, Staufen, Germany) under vigorous stirring at 8000 rpm, and homogenized for 2.5 min after complete addition. The antisolvent ratios (defined as “the volume ratio of organic phase to aqueous phase”) were 1:1, 1:2.5, 1:5, 1:10, respectively. After rotary evaporation (N-1300, Tokyo Riken Instruments Co., Tokyo, Japan), the ethanol and part of the water were removed until the solids content of the quercetin aqueous dispersion was 3% (*w*/*v*). In order to obtain dried quercetin pellets with different antisolvent ratios, the aqueous dispersion was freeze-dried for 72 h in a freeze-dryer (SCIENTZ-10N, Ningbo Xinzhi Biotechnology Co., Ltd., Ningbo, China).

### 2.3. Characterization of Quercetin Particles

#### 2.3.1. Visual Appearance

A certain amount of the aqueous dispersion of quercetin pellets at 1% (*w*/*v*) with different antisolvent ratios was taken and left in a transparent serum bottle to observe its appearance. The visual photos were taken by a cell phone (Apple 12, Apple Inc., Cupertino, CA, USA). The quercetin without antisolvent treatment at the same concentration was used as a control.

#### 2.3.2. Microstructural Observation and Estimated Size of Quercetin Particles

The microstructure of quercetin in the aqueous dispersions was studied using light microscopy (LM) with 40× objective (BMC-500, Phoenix Optics Co. Ltd., Jiangxi, China). 

The microstructure of the quercetin particles was also studied using atomic force microscopy (AFM). The aqueous dispersions of the native quercetin particles and the quercetin particles obtained by the antisolvent precipitation method were diluted to 0.01% (*w*/*v*), dropped onto freshly exfoliated mica sheets and dried naturally, and observed using AFM (Nanoscope IIIa, Bruker AXS, Santa Barbara, CA, USA) with a tapping mode.

The estimated size of the quercetin particles was manually measured over 50 particles from the AFM image using the microscopic image analysis software (Nano Measurer 1.2, Fudan University, Shanghai, China) [51,52,53]. As the quercetin particles are rod-like, the average of the measured diameters of over 50 particles was defined as the calculated diameter. 

#### 2.3.3. XRD

The crystallinity of the quercetin pellets was analyzed using X-ray diffraction (XRD) (Bruker AXS D8, Karlsruhe, Germany). The dried quercetin particles were placed on a glass sample table, flattened, and then placed on the bench for measurement. The scanning range was between 3° and 80° with a step size of 0.02°, and the subsequent analysis was performed using X-ray diffraction analysis software.

For a better comparison of the difference in crystallinity between the different samples, the relative crystallinity (%) was calculated as follows:

$$\mathrm{Relative}\;\mathrm{crystallinity}\;(\%)=\frac{A_{t}}{A_{0}}\times 100\%$$

where $A_{t}$ and $A_{0}$ are the area of the typic peak in the XRD curve for the quercetin obtained by antisolvent-treated and the native quercetin, respectively. 

#### 2.3.4. Contact Angle

In surfactant stabilized emulsions, the HLB is often used to characterize the hydrophilicity/hydrophobicity of the surfactant, whereas in the case of Pickering emulsions, the contact angle is used to characterize the wettability of particles, which is important for emulsion stabilization. In general, solid particles with contact angles less than 90° are considered hydrophilic, while those with contact angles greater than 90° are considered hydrophobic. The contact angle can be used to indicate changes in the surface properties of quercetin particles after antisolvent precipitation treatment and to determine whether the particles are stable as O/W emulsions or stable W/O emulsions [54]. The contact angle of the quercetin was measured by a contact angle analyzer (OCA 20 AMP, Data 118 physics Instruments, Germany). Next, 200 mg native quercetin pellets and lyophilized quercetin pellets treated by the antisolvent precipitation method were each pressed into 13 mm diameter and 2 mm thick slices using a tablet press; then, the slices were placed in an optical glass dish containing soybean oil, and 5 μL of water droplets were precisely released onto the sample surface using a syringe, and the droplet behavior images were captured using a camera and analyzed using SCA20 software for analysis. The above samples were measured twice in parallel, and each pressed tablet was titrated at least twice to minimize experimental errors.

### 2.4. Preparation and Characterization of Quercetin Pickering Emulsion

#### 2.4.1. Preparation of Quercetin Pickering Emulsion

The aqueous dispersions of the quercetin particles were diluted using 10 mM PBS (pH 7.0) to obtain aqueous dispersions with quercetin concentrations (*c*) of 0.5%, 1.0%, 1.5%, 2.0%, 2.5%, and 3% (*w*/*v*). Subsequently, the quercetin dispersions were mixed with soy oil at different ratios. The mixture was homogenized for 2 min using a T25 high-speed homogenizer (IKA, Staufen, Germany) at 10,000 rpm to obtain the emulsions. All of the emulsions were then immediately sealed and stored at room temperature.

#### 2.4.2. Visual Photos

The series samples were left to stand for 7 days, and their appearance was photographed at 0 and 7 days to observe the change condition of their appearance.

#### 2.4.3. Microstructure and Particle Size Analysis

The microstructures of various quercetin emulsions were evaluated by light microscopy (LM) using a digital microscope (BMC-500, Phoenix Optics Co. Ltd., China) at 0 and 7 days, respectively. For the LM observations, the emulsion was gently dropped onto a slide and observed without a coverslip using a 10× objective. If necessary, dilute the emulsion with deionized water for clearer observation.

The droplet size of emulsions at 0 and 7 days was measured using a laser diffraction particle size analyzer (SALD-2300, Shimadzu Corporation, Kyoto, Japan).

### 2.5. Statistical Analysis

The data statistics are expressed as mean ± standard deviation and combined with graphical analysis using SPSS and origin 2018 software.

## 3. Results and Discussion

### 3.1. Physicochemical Properties of Quercetin Dihydrate Particles

**Figure 1:**
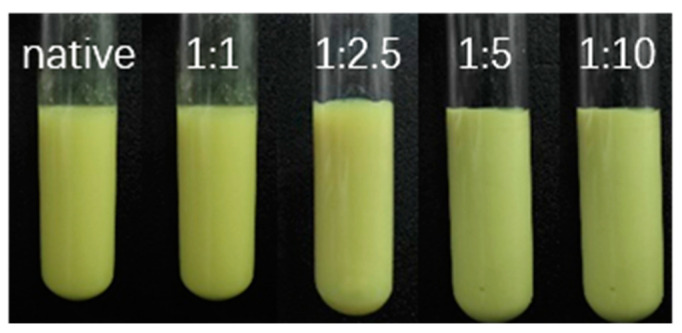
Water dispersions of native quercetin and quercetin particles (*c* = 1%) formed by antisolvent precipitation under various solvent/antisolvent ratios.

The quercetin pellet dispersions were obtained using the antisolvent treatment method, followed by rotary evaporation to remove solvents and part of the antisolvent (see Figure 1). As water and ethanol are highly acceptable in food, water was chosen here as the antisolvent and ethanol as the solvent [30]. As seen in Figure 1, both the native quercetin and the antisolvent-treated quercetin could be dispersed in water to form an opaque, slightly yellow suspension, indicating that quercetin may exist in a larger particulate form. Similar results have been reported in many other particles using the same method, including zein particles, phytosterol particles, and curcumin particles [30,32,55].

**Figure 2:**
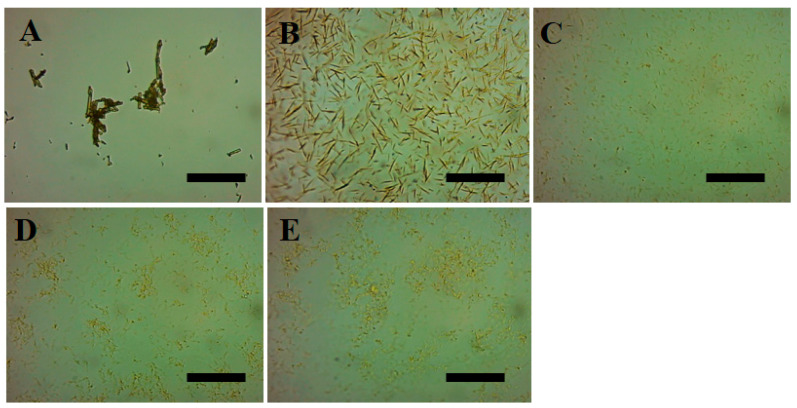
Light microscopic images of native quercetin particles dispersion (**A**) and quercetin particles dispersion formed by antisolvent precipitation with various antisolvent ratios ((**B**), 1:1; (**C**), 1:2.5; (**D**), 1:5; (**E**), 1:10). The scale bar is 50 μm.

The antisolvent treatment significantly changed the particle morphology and size of the quercetin. The optical microscopy results of the particle dispersions (Figure 2) showed that the native quercetin was present as aggregated coarse rod-like crystals up to several tens of microns in size (see Figure 2A). This is similar to the results (e.g., 50.1 μm) reported in the literature [56]. After the antisolvent treatment (Figure 2B–E), the size of the quercetin was significantly reduced and more uniformly dispersed. Specifically, it appears that the 1:1 quercetin shows a needle-like morphology with length of about 10 μm, and the 1:2.5, 1:5, and 1:10 quercetin are smaller in size, but have some degree of aggregation. The size of the particles was closely related to the process of antisolvent treatment which includes nucleation and crystal growth. The greater the formation of nucleation and the slower the crystal growth, the smaller the particle size. As a result, the final size of the particles remains unchanged due to the crystal stop growth at certain stages, which depends on particle-particle interactions. Therefore, the size of the particles obtained from the antisolvent treatment are always smaller than the native particles when dispersed in water. Increasing the antisolvent ratio further decreases the size, which is mainly due to retarded crystal-growth and aggregation.

**Figure 3:**
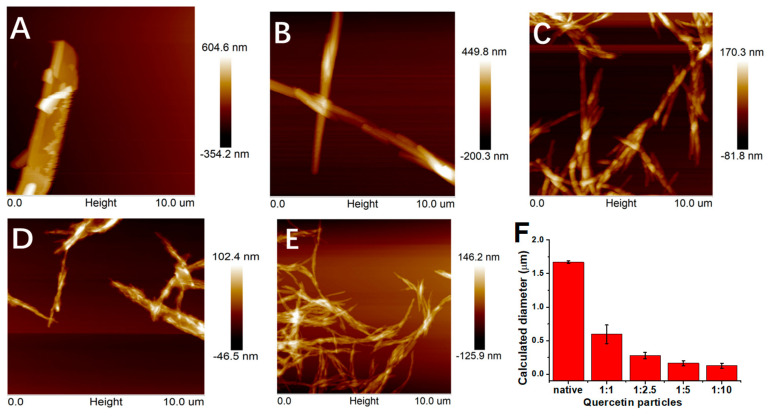
Atomic force microscopic images (**A**–**E**) and calculated diameter (**F**) of native quercetin (**A**) and quercetin particles formed by antisolvent precipitation with various antisolvent ratios ((**B**), 1:1; (**C**), 1:2.5; (**D**), 1:5; (**E**), 1:10).

Atomic force microscopy (AFM) was used to observe the detail of the morphological changes of the quercetin particles after the antisolvent treatment (see Figure 3A–E). The size (diameter) of the quercetin was estimated based on the AFM images using the software (Nano measurer 1.2), and the results are shown in Figure 3F. As shown in Figure 3, the native quercetin presents a very large rod shape, with a diameter (the smallest unit) of about 1.7 μm. After the antisolvent treatment, the diameter of the quercetin decreases significantly, e.g., the diameter of quercetin (antisolvent ratio, 1:1) is about 0.6 μm. Increasing the antisolvent ratio to 1:10, there is a further decrease in the quercetin particle diameter (0.13 μm), but the degree of aggregation increases. The AFM results are well in agreement with the optical microscopy results. The decrease in the particle size due to the increase in the antisolvent ratio is common in the literature, which is mainly due to the self-assembly process (nucleation-crystallization growth) of the substance [56,57]. The increase in the degree of aggregation is mainly due to an increase in the interparticle interactions, suggesting that the antisolvent treatment may have altered the surface properties of the quercetin particles. As a result, the antisolvent treatment plays two roles in regard to quercetin: it decreases the size (the diameter of minimum unit) of quercetin and it changes the surface properties of quercetin, which enhances the interactions between neighboring quercetin particles. 

### 3.2. Crystallinity and Contact Angle of Quercetin Particles

**Figure 4:**
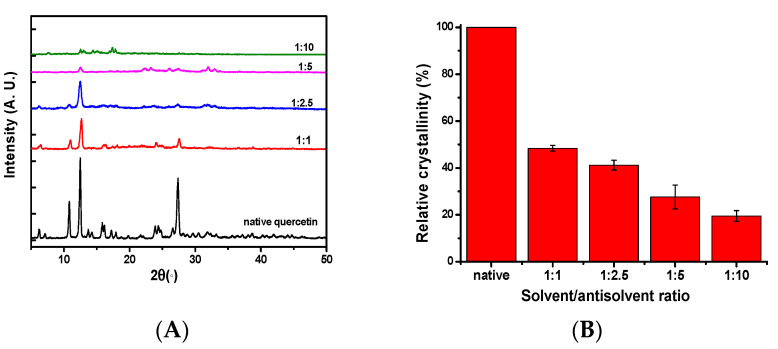
XRD (**A**) and relative crystallinity (%) (**B**) of native quercetin and quercetin particles formed by antisolvent precipitation with various antisolvent ratios (1:1, 1:2.5, 1:5, 1:10).

The effect of the antisolvent treatment on the crystalline nature of quercetin was evaluated using the XRD technique. As shown in Figure 4A, the XRD spectra at 10–30° showed a series of sharp peaks, indicating the crystalline nature of quercetin. The typic peaks of the native quercetin are mainly at 2θ of 6.3–7.3°, 10.8°, 12.5°, 13.6–14.3°, 15.7–18.0°, 24.4°, and 27.3°, which represented the crystalline part of quercetin [25]. Compared with the native quercetin, the quercetin particles obtained by antisolvent precipitation have the same diffraction peaks of 12.5°, 13.6–14.3°, 24.4°, and 27.3°, but have almost no diffraction peaks of 6.3–7.3°, 10.9°, and 15.7–18.0°. Moreover, compared to the native quercetin, the intensity of the same diffraction peak of quercetin is also reduced significantly after antisolvent precipitation and further reduced upon increasing the antisolvent ratio from 1:1 to 1:10. The decline in the peak intensity indicated a decrease in crystallinity. The decrease in the crystallinity could be further quantitatively evaluated by the relative crystallinity calculated from the XRD spectra. As shown in Figure 4B, the order of the relative crystallinity between the different quercetin were as follows: native (100%) > 1:1 (48.1%) > 1:2.5 (41.18%) > 1:5 (27.62%) > 1:10 (19.52%). The decrease in the crystallinity indicates an increase in the degree of the particles’ amorphousness. Much of the literature has also reported that the crystallinity decreases after antisolvent treatment, such as in curcumin and phytosterol [30,32]. The change in the crystallinity and morphology of quercetin may be accompanied by a change in its surface properties (e.g., wettability) [32]. 

The hydrophilic/hydrophobic characteristics (wettability) of particles can be reflected by the contact angle (θ). To evaluate the effect of the antisolvent treatment on the face wettability of quercetin particles, the contact angle of the quercetin particles was measured, and the results are summarized in Table 1. From Table 1, it can be seen that the contact angle of the native quercetin is 102.65°, which is slightly hydrophobic. This value is slightly higher than that of the quercetin reported by Zembla et al., which is 89.9 at pH 3.0 and 79.1 at pH 7.0 [18]. This discrepancy may be due to the use of different methods to obtain the contact angle. In that work, the contact angles of the aqueous phase and oil phase were measured, and the three-contact angle was calculated by Young’s equation, whereas in our work, the three-contact angle is directly measured. Another possible reason is that the material used is different (quercetin vs. quercetin dihydrate). After the antisolvent treatment, the contact angle of quercetin decreased significantly (84.85–65.15°), and further reduced upon increasing the antisolvent ratio from 1:1 to 1:10. The decrease in the contact angle indicates the enhanced hydrophilicity of the particles, which would also help quercetin to partition better between the oil and water phases. 

### 3.3. Microstructure and Particle Size of Quercetin Particle-Stabilized Emulsions

#### 3.3.1. Effect of Solvent/Antisolvent Ratio

**Figure 5:**
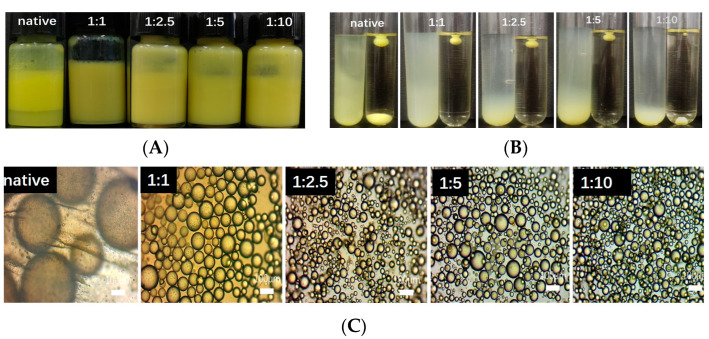
(**A**) Visual appearances of emulsions stabilized by native quercetin and antisolvent-treated quercetin at antisolvent ratios of 1:1, 1:2.5, 1:5, 1:10; (**B**) Droplet test results of emulsions stabilized by native quercetin and antisolvent-treated quercetin at antisolvent ratios of 1:1, 1:2.5, 1:5, 1:10; (**C**) Optical microscopy images of emulsions stabilized by native quercetin and antisolvent-treated quercetin at antisolvent ratios of 1:1, 1:2.5, 1:5, 1:10. In Figure 5B, emulsion was dropped into pure water (left tube) and pure oil (right tube), respectively. The emulsions were stabilized by 1% quercetin particle and the oil fraction is 0.5. The scale bar is 100 μm.

We first evaluated the effect of the antisolvent ratio on the properties of the quercetin emulsions. The appearance, types, and microstructures of the quercetin-stabilized emulsions with different antisolvent ratios are shown in Figure 5. As can be seen in Figure 5A, when soybean oil was utilized as the oil phase, all of the quercetin particles created pale yellow emulsions. All of the emulsions were stable against creaming, with the exception of the native quercetin-stabilized emulsion. Due to the solid particulate nature (crystals), the emulsions stabilized by quercetin particles with or without antisolvent treatment could be considered as forms of Pickering emulsions. The drop test (Figure 5B) showed that the emulsions formed were all oil-in-water emulsions, which is basically consistent with the contact angle data of the quercetin particles. This is consistent with the previous study by Wang et al. (oil-in-water type), but different to that by Zembyla et al. (water-in-oil type). The quercetin used in the current study was in the form of dihydrate and was pre-dispersed in the water phase before emulsification, which could explain the different results. This would also provide an explanation for the native quercetin with a contact angle of >90° still forming an oil-in-water emulsion. These results also show that, in addition to the contact angle, the pre-location before emulsification could also greatly influence the emulsion properties. The microstructure results (Figure 5C) showed that the particle size of the native quercetin stabilized emulsion was very large (~350 μm), which also explained its easy delamination. Compared with the native quercetin, the particle size of the antisolvent-treated quercetin-stabilized emulsion was significantly reduced (30–50 μm), and the particle size of the emulsion decreased slightly with the increase in the proportion of the antisolvent. The microstructures of the emulsions also clearly confirm the above results of the droplet size. Similar results have been found in other emulsions stabilized by phytosterol particles and curcumin particles [30,32]. The above results indicated that the antisolvent treatment significantly improved the emulsifying ability of the quercetin particles. Combined with the above results of the particles’ characteristics, the improvement in the quercetin’s emulsifying ability after the antisolvent treatment could be mainly attributed to its better surface wettability and decreased particle size. On one hand, the antisolvent treatment reduced the particle size of the quercetin, which are favorable for emulsion stabilization. On the other hand, the antisolvent treatment decreased the contact angle apart from 90°, which is unfavorable for emulsion stabilization. Moreover, in addition to the improvement in the emulsion’s physical stability, the quercetin particles at the interface are also expected to improve the oxidation stability of the emulsions due to its antioxidative nature. 

#### 3.3.2. Effect of Particle Concentration

**Figure 6:**
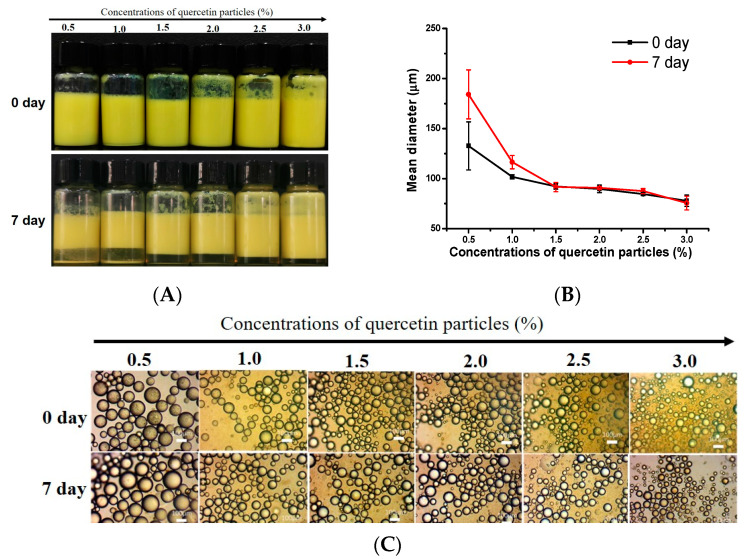
The effect of particle concentrations on visual appearances (**A**), mean diameter (**B**) and optical microscopy images (**C**) of emulsions prepared by antisolvent quercetin particles (the antisolvent ratio of quercetin particles was 1:1, *c* = 0–3%) after storage for day 0 and day 7. The ratio of oil/water of the emulsion is 1:1. The scale bar is 100 μm.

The effect of the particle concentration on the properties of the emulsions stabilized by the antisolvent-treated quercetin (antisolvent ratio is 1:1) is shown in Figure 6. As can be seen in Figure 6A, the freshly made quercetin emulsions all had a light-yellow color and had begun to cream somewhat after seven days of storage. The extent of the creaming gradually reduced upon increasing the particle concentration up to 2.0%, and remained unchanged when further increasing the particle concentrations. Particle-stabilized emulsions frequently experience this creaming phenomenon, which is primarily caused by the large size of the emulsion droplet obtained by low-energy emulsification technology. As shown in Figure 6B, with increasing the particle concentration from 0.5 to 1.5%, the droplet size of the emulsion stabilized by quercetin obtained at a 1:1 antisolvent ratio decreased significantly from 130 μm to 95 μm, while further increases in the particle concentration to 3% resulted in a decrease in size, to 80 μm. At a specific oil fraction (e.g., 0.5), when the particle concentration is increased, more particles are available to stabilize the oil-water interface, thus facilitating the formation of smaller emulsion droplets. Moreover, the microstructure of the emulsions (Figure 6C) shows that the particle size decreases as the particle concentration increases, confirming the particle size data above. With the exception of the phenomenon of creaming, no oil was released on the top of the emulsion layer after seven days of storage. Additionally, a slight increase in the emulsion size occurred at lower concentrations, but no further changes in the emulsion size occurred at higher concentrations, indicating that the quercetin emulsions (especially at higher concentrations) have high coalescence stability during storage. Pickering emulsions, which have been widely reported in the literature, are known for their remarkable stability against coalescence [2,5,14,58].

#### 3.3.3. Effect of Oil Fraction

**Figure 7:**
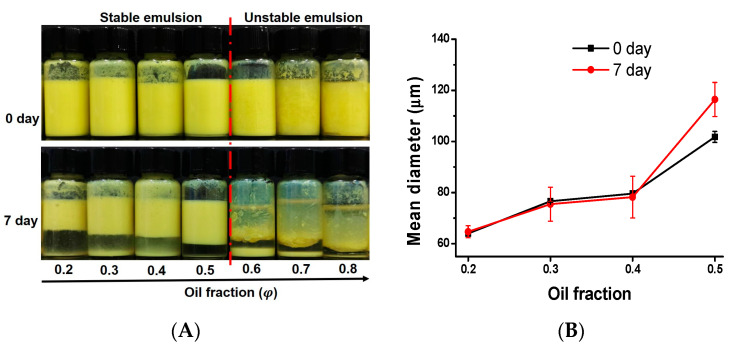
Visual appearances (**A**) and droplet size (**B**) of emulsions prepared by antisolvent quercetin particles (the antisolvent ratio of quercetin particles was 1:1, *c* = 1%) at various oil fractions after storage for 0 day and 7 days.

The effect of the oil fraction on the particle size and stability of the quercetin emulsions was evaluated, and the results are shown in Figure 7. As shown in Figure 7A, with soybean oil as the oil phase, 1% of the quercetin particles (antisolvent ratio is 1:1) could form emulsions with an oil fraction of 0.2–0.5, and above 0.5 (oil fraction) no stable emulsion was formed. The formed emulsions, after being stored for seven days, all underwent different degrees of creaming, which is similar to those of the emulsions formed at various concentrations of quercetin particles. As the oil fraction (*φ*) increased (from 0.2 to 0.5), the emulsion layer (upper layer) gradually increased, while the height of the lower layer (aqueous layer) gradually decreased. At a fixed particle concentration in the aqueous phase, the total volume of the emulsion droplets formed increases as the oil fraction increases, and thus the emulsion layer also increases. The changes in the droplets’ size at various oil fractions on days zero and seven are shown in Figure 7B. As shown in Figure 7B, when the oil phase ratio is increased from 0.2 to 0.4, the emulsion particle size increases slowly from 62 μm to 78 μm. Further increasing the oil phase ratio to 0.5, the emulsion particle size increases significantly to 100 μm. The increase in the emulsion size is mainly a result of the increased interfacial area and droplet volume. Moreover, upon increasing the oil fraction, the amount of quercetin particles used to stabilize the oil-water interface are reduced, resulting in a lack of sufficient particles to cover the newly formed oil-water interface. As a result, a certain amount of droplet coalescence and the droplet size increased. After seven days of storage, the droplet size of the emulsion remained unchanged at an oil fraction of 0.2–0.4. and slightly increased at an oil fraction of 0.5. The results suggested that the emulsions stabilized by the 1% quercetin obtained by antisolvent treatment (1:1) with an oil fraction of 0.2–0.4 showed high stability against coalescence. 

## 4. Conclusions

The antisolvent treatment can transform quercetin into a good oil-in-water Pickering emulsion stabilizer. The antisolvent treatment could significantly change the characteristics of quercetin particles, including a reduced size, crystallinity, and contact angle, which is strongly influenced by the antisolvent ratio. Compared with native quercetin, the ability of antisolvent-treated quercetin to stabilize emulsions and its emulsion stability against coalescence were significantly improved. The properties of quercetin-stabilized emulsions can be modulated by changing the antisolvent ratio, quercetin particle concentration, and oil fraction. The results of this paper are instructive for the development of highly stable emulsions, as well as novel quercetin functional foods.

## Figures and Tables

**Table 1:** Contact angle (θ) of native quercetin and quercetin particles formed by antisolvent precipitation with various antisolvent ratios (1:1, 1:2.5, 1:5, 1:10).

Samples	Native	1:1	1:2.5	1:5	1:10
Contact angle (°)	102.65 ± 0.64 ^a^	84.85 ± 0.21 ^b^	75.63 ± 0.78 ^c^	69.90 ± 2.62 ^d^	65.15 ± 1.48 ^e^

The contact angles were determined on quercetin tablets by sessile drop method. The letter a–e represented the significant statistical differences (*p* < 0.05) between different quercetin particles.

## Data Availability

Data is contained within the article.
